# Supplementary material for: Seminoma and Embryonal Carcinoma Footprints Identified by Analysis of Integrated Genome-Wide Epigenetic and Expression Profiles of Germ Cell Cancer Cell Lines
Source: PLoS One. 2014 Jun 2;9(6):e98330. doi: 10.1371/journal.pone.0098330 (PMC4041891; doi:10.1371/journal.pone.0098330)
Supplement: File S1 — ZIP file containing DMRforPairs output for significant regions. Please start from the html files. (ZIP) [file pone.0098330.s008.zip › figures/5238.pdf]

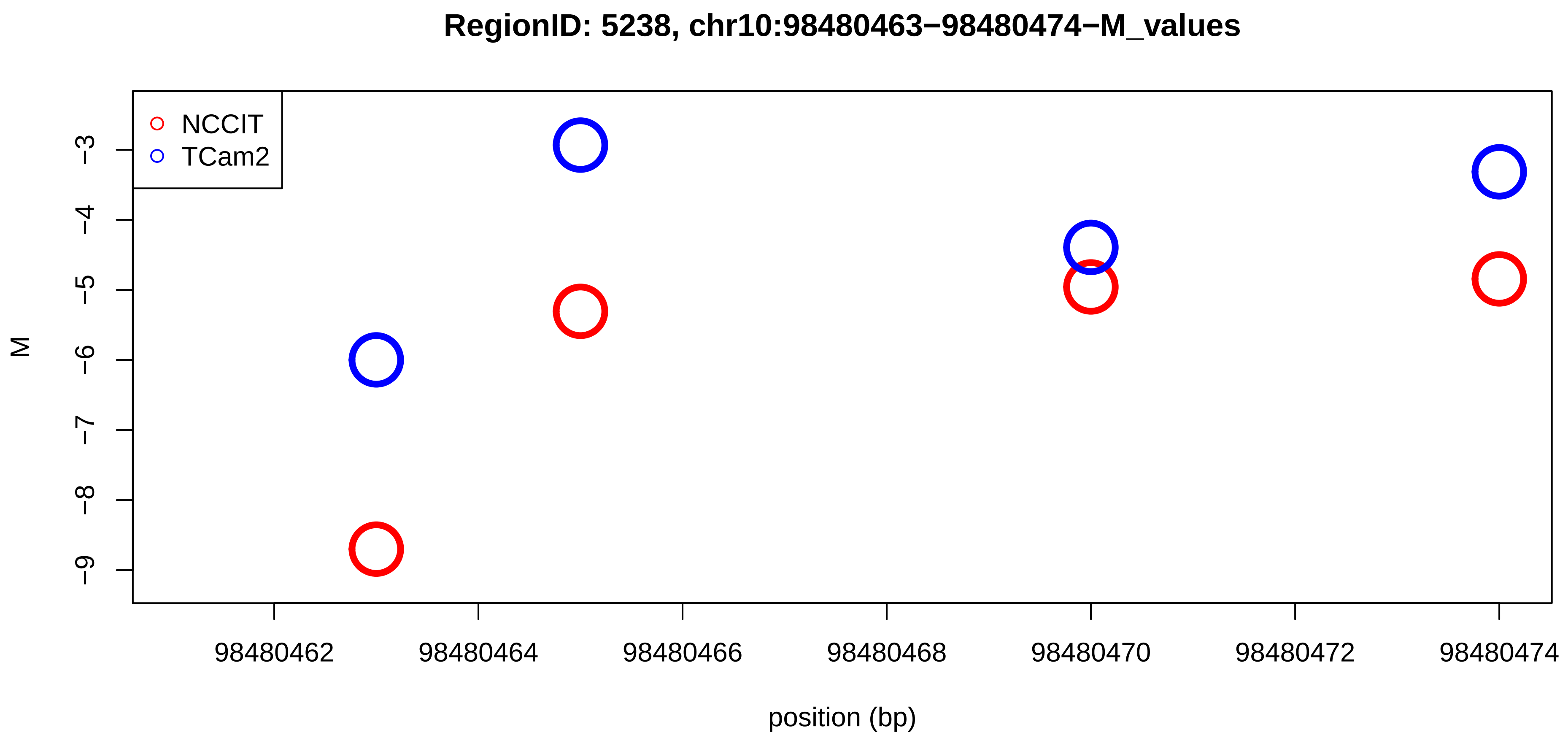
RegionID: 5238, chr10:98480463−98480474−M_values
NCCIT
TCam2
M
−3
−4
−5
−6
−7
−8
−9
98480462
98480464
98480466
98480468
98480470
98480472
98480474
position (bp)

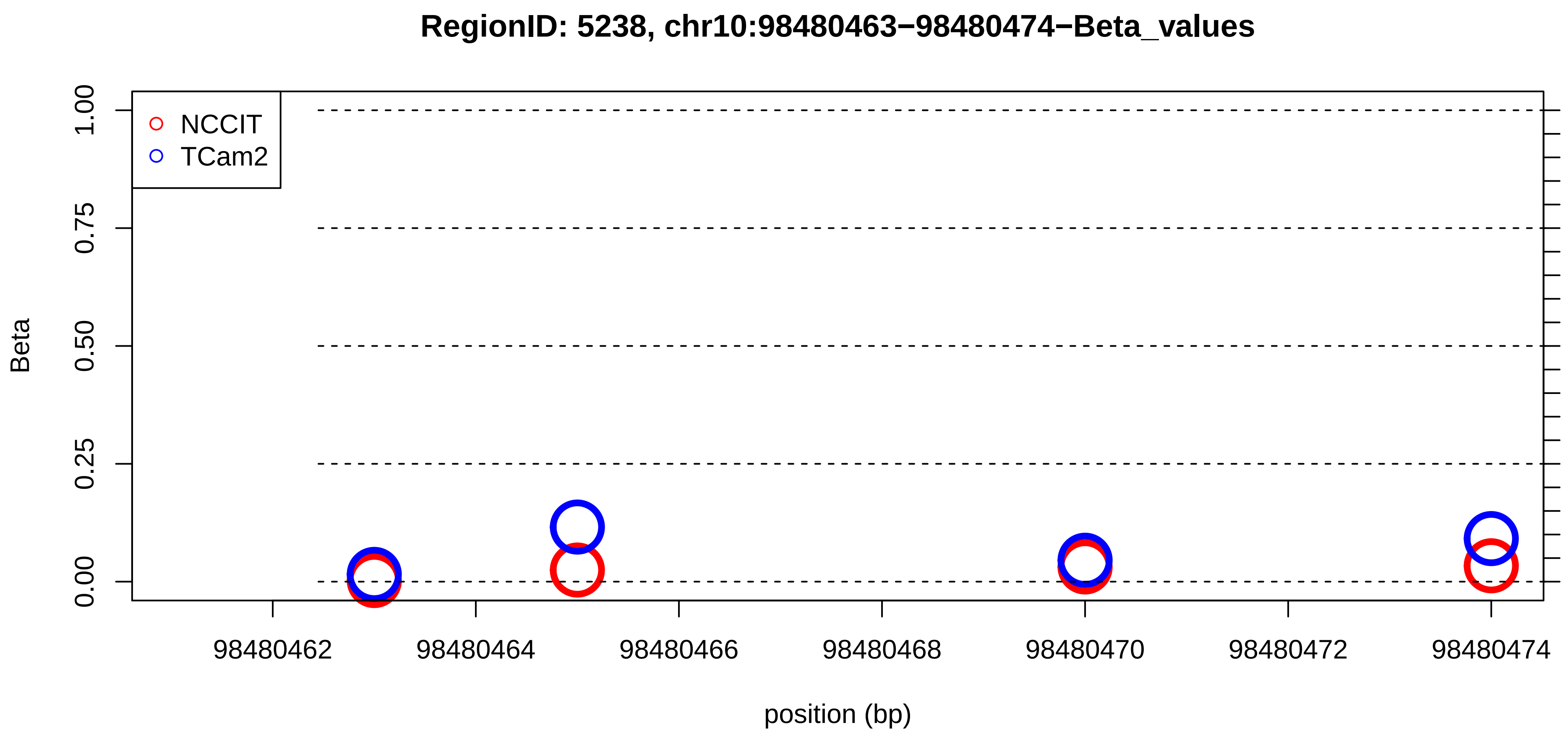
RegionID: 5238, chr10:98480463−98480474−Beta_values
NCCIT
TCam2
Beta
1.00
0.75
0.50
0.25
0.00
98480462
98480464
98480466
98480468
98480470
98480472
98480474
position (bp)
